# A Generalized Robot Navigation Analysis Platform (RoNAP) with Visual Results Using Multiple Navigation Algorithms

**DOI:** 10.3390/s22239036

**Published:** 2022-11-22

**Authors:** Chuanxin Cheng, Shuang Duan, Haidong He, Xinlin Li, Yiyang Chen

**Affiliations:** 1 School of Mechanical and Electrical Engineering, Soochow University, Suzhou 215031, China; 2 Department of Digital Media, Soochow University, Suzhou 215031, China

**Keywords:** analysis platform, robot navigation, algorithm testing

## Abstract

The robotic navigation task is to find a collision-free path among a mass of stationary or migratory obstacles. Various well-established algorithms have been applied to solve navigation tasks. It is necessary to test the performance of designed navigation algorithms in practice. However, it seems an extremely unwise choice to implement them in a real environment directly unless their performance is guaranteed to be acceptable. Otherwise, it takes time to test navigation algorithms because of a long training process, and imperfect performance may cause damage if the robot collides with obstacles. Hence, it is of key significance to develop a mobile robot analysis platform to simulate the real environment which has the ability to replicate the exact application scenario and be operated in a simple manner. This paper introduces a brand new analysis platform named robot navigation analysis platform (RoNAP), which is an open-source platform developed using the Python environment. A user-friendly interface supports its realization for the evaluation of various navigation algorithms. A variety of existing algorithms were able to achieve desired test results on this platform, indicating its feasibility and efficiency for navigation algorithm analysis.

## 1. Introduction

Mobile robots play a significant role in people’s daily life in areas such as autonomous cleaning [1], structural inspection [2], logistics transportation [3], and more. As the basic function allowing mobile robots to execute various instructions, navigation technology which requires robots to move safely to designation has attracted the attention of researchers in the robotics community [4,5]. With advances in computer hardware performance, increasing numbers of algorithms are being applied in mobile robot navigation [6], such as rapidly-exploring random tree (RRT) [7], simultaneous localization and mapping (SLAM) [8], artificial potential field method [9], and fuzzy logics [10]. These algorithms have all achieved satisfactory robot navigation results to a certain extent [11,12].

A massive number of experiments may be required for the success of the above-mentioned navigation algorithms; however, there are drawbacks when testing algorithms in practical environments at an early stage. First, various unstabilizing factors exist in reality which may affect the success of the navigation results [13]. For instance, the failure of certain components can affect the overall performance, while disturbances in the environment can degrade the accuracy of data collection [14]. These negative effects may lead to the failure of algorithm implementation. For these reasons, it can be confusing to try to make a preliminary judgment as to whether the algorithm is effective when applying it in practice. In addition, it takes a period of time to load the program into the robot for each test. It is common knowledge that an algorithm requires debugging in order to achieve a desired result, and each modification needs to pass through the results verification process. It is not recommended to modify and test the program using a physical robot because of loading time consumption. Last but not least, especially for learning algorithms, massive collisions with obstacles are largely inevitable in the early stage, and can result in serious machine damage. Accordingly, modelling and simulation are presently significant parts of the engineering experiment processes, especially for robotic systems.

Analysis platforms are widely used in robotics, and underpin the advanced research in this field [15]. Multiple open-source and proprietary robot modeling and analysis platforms have been developed with advancements in affordable and potent computing technology [16]. There are currently several simulation and analysis environments, including Webots [17], Gazebo [18], and V-rep [19]. Gazebo is a powerful open source physical simulation environment usually utilized to test various algorithms for both wheeled and legged robots, as shown in [20,21]. It supports a variety of high-performance physics engines and contains many different architecture models, offering the ability to accurately and effectively simulate real scenes. However, it is cumbersome to configure the simulation environment, meaning that that abecedarians may not wish to run their algorithms on it. Furthermore, loading various 2D or 3D graphics consumes computing resource, which seriously reduces the operating efficiency of the tested algorithms, especially those requiring the use of GPU acceleration. Compared with Gazebo, Webots is supported by complete documentation and examples for reference, and as such can be used with low learning cost. Nevertheless, the visualization of Webots is not outstanding, and data cannot be automatically saved during the analysis process. V-rep is famous for integrating various robotic arms and a few common mobile robots whose control mode can be directly modified. However, the parameters of the model components are concentrated on an entire model tree, which adds inconvenience to the operation. Taking a mobile robot as an example, the parameters for its wheels cannot be selected and modified directly in V-rep.

There is no doubt that the simulation and analysis platforms mentioned above are both useful and powerful. However, a simulation scene similar to the real environment is required before analysis, which seems difficult for most abecedarians. In addition, the analysis process requires sufficient computing power, otherwise the operation speed becomes surprisingly slow. Hence, researchers have sought to develop suitable platforms for their special requirements. For example, an automatic creation of simulator for the normal Evolutionary Robotics process was introduced in [22,23]. For path planning and obstacle avoidance algorithms, rendering architectural graphics is not necessary at all. The key of obstacle avoidance is not the authentic reproduction of obstacles, only how to avoid them. Rendering graphics uses computing resources to extend the time of testing algorithms, which is not conducive to initial judgment of the effectiveness of obstacle avoidance algorithms because of their complex scenes. Accordingly, a simple and reliable analysis platform is more conducive to testing and finding applicable path planning and obstacle avoidance algorithms.

In this paper, a robot navigation analysis platform named RoNAP is developed using the Pygame package in the Python environment. Compared to existing platforms, RoNAP’s remarkable features are its complete functions and simple operation. On the one hand, it has the ability to construct a scene containing obstacles and a test robot, providing a neat and narrow storage environment in the modern logistics industry. Conventional sensors are equipped to detect surroundings to obtain the required information. RoNAP provides a clear visual interface in which the movement state and path of the robot are observed in real time. The data generated at each epoch are recorded. These functions are fully sufficient to support the complete analysis of a navigation algorithm. On the other hand, to reduce the computer configuration requirements, extra parts such as 3D graphics support are not loaded, allowing it to be realized with only a CPU. Moreover, the design of the interactive interface is simple, and the underlying algorithm is written in blocks. Anyone with knowledge of basic Python programming is capable of performing algorithm testing using RoNAP.

The rest of this paper is organized as follows: Section 2 introduces the functions and advantages of RoNAP, and the implementation mechanisms of a number of functions are described in detail; Section 3 plays the role of the simulator’s operation manual; Section 4 presents several navigation algorithm experiments completed on the platform, which prove that RoNAP is capable of analyzing various mobile robot navigation algorithms; finally, in Section 5, the work is summarized and possibilities for future work are discussed.

## 2. Framework Structure

RoNAP mainly employs Pygame packages to complete the creation of various visual interfaces. In this section, its abundant functions and prominent characteristics are introduced in detail.

### 2.1. Graphic User Interface

**Figure 1:**
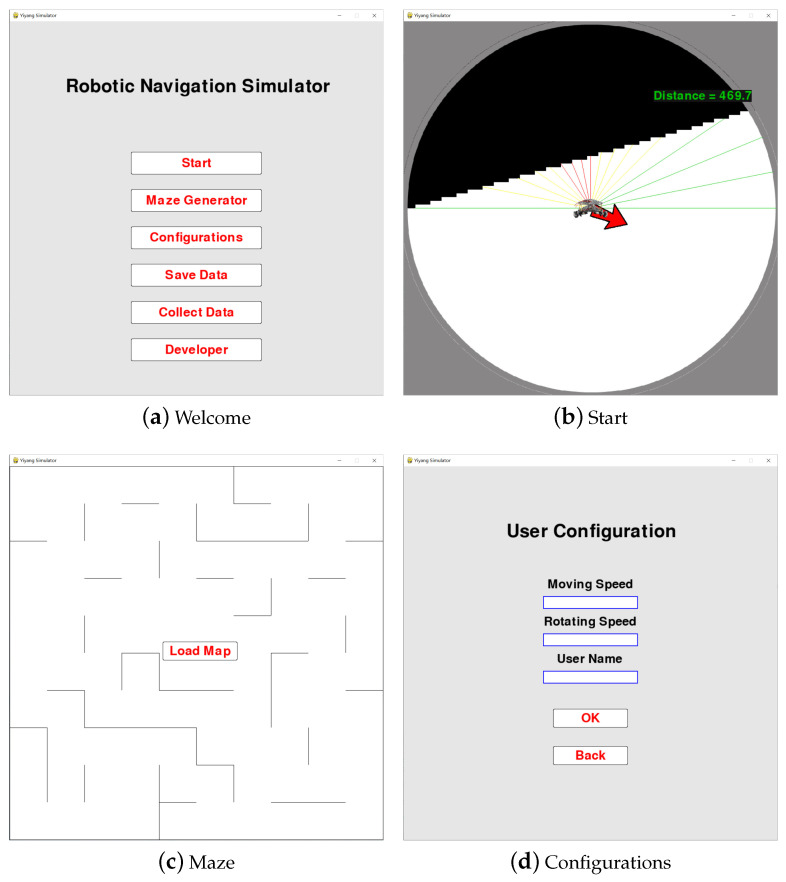
RoNAP provides several visual interfaces for user operation. Subfigure (**a**) shows the initial interface, with six functions for robot navigation analysis available by clicking on corresponding button. Subfigure (**b**) shows the start interface, where scene information within the detectable range of the laser can be observed. Subfigure (**c**) shows the maze interface, where a new scene with random obstacles is generated and loaded as a picture. Subfigure (**d**) shows the user configuration interface, where movement speed, rotation speed, and user name can be modified.

As shown in Figure 1a, there are six buttons in the initial interface, which respectively represent the six functions of this platform. By clicking on different buttons, a corresponding interface is provided to realize different functions. These functions are described as follows:

1. **Start:** This function button offers an interface which displays the real-time motion pose of the test robot, as shown in Figure 1b. This interface is built from the first-person perspective of the robot; the scene within the detectable range of the robot is provided, which changes as the robot moves. In addition, the distance of laser detection is indicated by different color rays; the red arrow directly points to the destination position, while the green digits on the upper right indicate the distance to the destination position.
2. **Maze Generator:** This function button is used to generate a new scene with randomly placed obstacles, as shown in Figure 1c. When the user clicks on “Load Map”, the new scene map is loaded as a background picture for the next robot navigation analysis.
3. **Configurations:** This function allows users to modify related parameters, such as movement speed, rotation speed, and user name. As shown in Figure 1d, when filling in something with correct format in the blank, the initial settings are replaced.
4. **Save Data:** This function button is able to save the data generated during the analysis process, including the laser detection distance, distance to the destination position, the direction in which the robot is moving, the direction to the destination, and the action performed. All information is saved in a .csv file named using the format “username + usage date”.
5. **Collect Data:** This function button offers an interface similar to the **Start** button, where the movement of robots is controlled by the keyboard. Human policy-making data are collected in the scene.
6. **Developer:** This function button provides related information on the developer, which allows academics interested in the same field to contact the developer for further communication and learning.

### 2.2. Features of RoNAP

**Figure 2:**
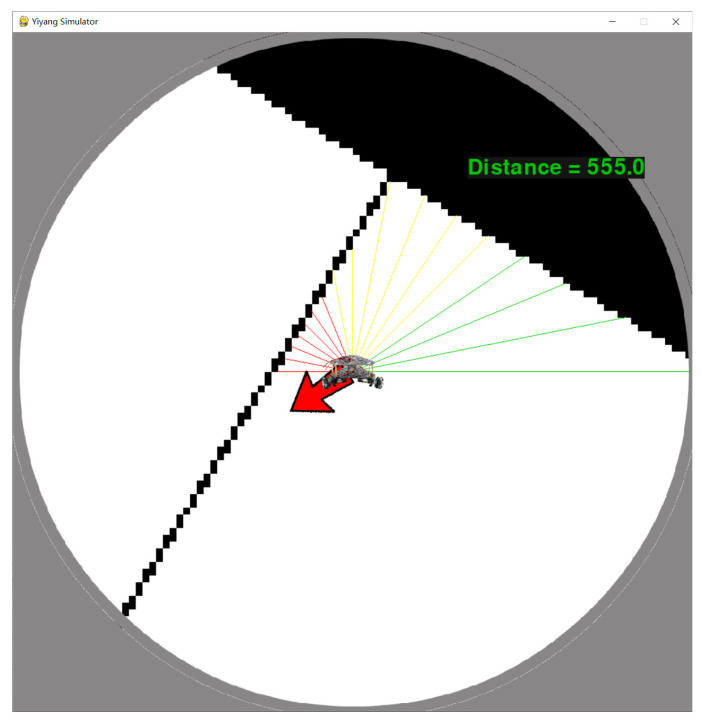
The main simulation interface of RoNAP, in which the scene information around the mobile robot is displayed from the first-person perspective.

In order to clearly observe the movement pose of the robot, RoNAP provides a clear first-person perspective interface, as shown in Figure 2. The field of view is focused on the circular area around the robot, and the radius is the maximum detection distance of the laser. In this interface, the mobile robot remains stationary, meaning that the emission angle of each laser does not change, which is convenient for calculating the distance between obstacles and the robot. Meanwhile, a rotating scene $\theta_{s}$ is provided by changing the robot’s forward angle *z* to ensure that the orientation of the robot relative to the scene conforms to the actual change, where

(1) $$\theta_{s}=90-z.$$

**Figure 3:**
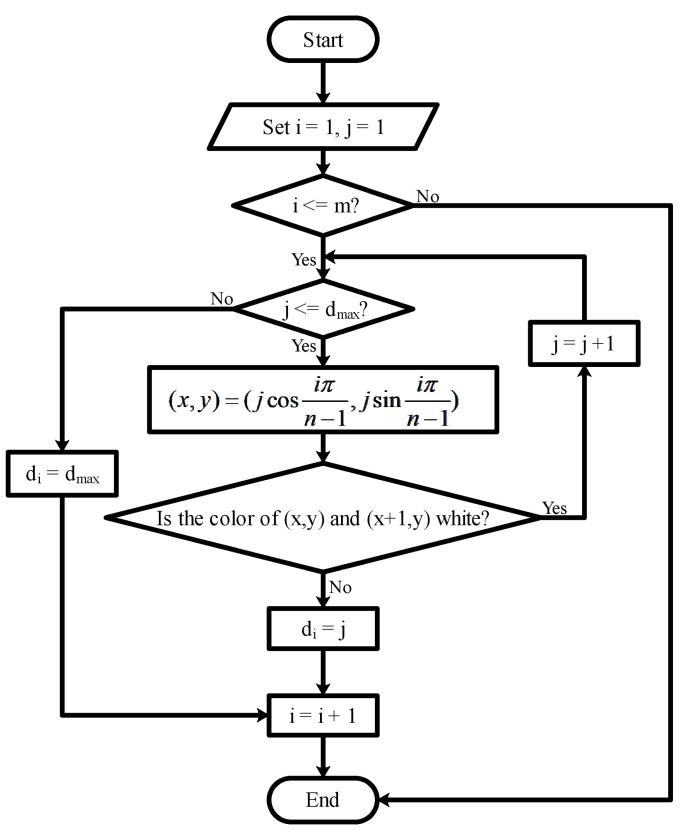
The complete mechanism flow utilized to generate laser ranging information in RoNAP.

This platform offers several sensors to obtain scene information. The laser is set to utilize *m* beams to detect obstacles in the direction of movement. Whether the laser reaches the obstacle is determined according to the color of the coordinate position on the picture, as described in Figure 3. Each distance is denoted as $d_{i}$, i=0,…,m, and the distance detected by the lasers is indicated by rays with different colors, as follows:

(2) $$c_{i}=\left\{\begin{array}{c} \begin{array}{cc} \mathrm{Green}, & \;0.6d_{max}<d_{i}⩽d_{max},\\ \mathrm{Yellow}, & \;0.3d_{max}<d_{i}⩽0.6d_{max},\\ \mathrm{Red}, & \;\;\;\;\;\;\;\;\;\;0<d_{i}⩽0.3d_{max}. \end{array} \end{array}\right.$$

The detection coverage is $\left[0^{\circ },\;180^{\circ }\right]$, which is capable of fully perceiving all obstacles ahead of the robot.

Furthermore, a location sensor is incorporated in the design to obtain the current position (x,y) and destination position $\left(x^{d},y^{d}\right)$. The distance to the destination is computed by

(3) $$D=\sqrt{{\left(x-x^{d}\right)}^{2}+{\left(y-y^{d}\right)}^{2}}.$$

This distance is displayed on the **Start** interface to indicate whether the robot is moving towards the destination position. The sign indicating whether the final destination position has been reached is determined by

(4) D<ϵ,

where ϵ is the acceptable distance error; at this point, “TaskComplete” is printed to remind the user. After obtaining the coordinates of the start position and destination position, the angle between the line of the two points and the x-axis is computed by

(5) $$\theta =\left\{\begin{array}{c} \begin{array}{cc} \frac{180}{\pi }\cdot \arctan\frac{y-y^{d}}{x-x^{d}},\;x_{d}>x, & \\ \frac{180}{\pi }\cdot \arctan\frac{y-y^{d}}{x-x^{d}}+180,\;x_{d}<x, & \\ 90,\;\;\;\;\;\;\;\;\;\;\;x^{d}=x\;\&\;y^{d}>y, & \\ 270,\;\;\;\;\;\;\;\;\;\;\;x^{d}=x\;\&\;y^{d}<y. \end{array} \end{array}\right.$$

The direction to the destination is shown by the red arrow in Figure 2, which rotates with the scene based on $\theta_{a}$, where

(6) $$\theta_{a}=90+\left(\theta -z\right)\%\;360,$$

and % denotes the remainder operation. Furthermore, a gyroscope is equipped to measure the direction of the robot’s movement *z*. The combination of all the above-mentioned information is enough to determine the pose of the robot.

**Figure 4:**
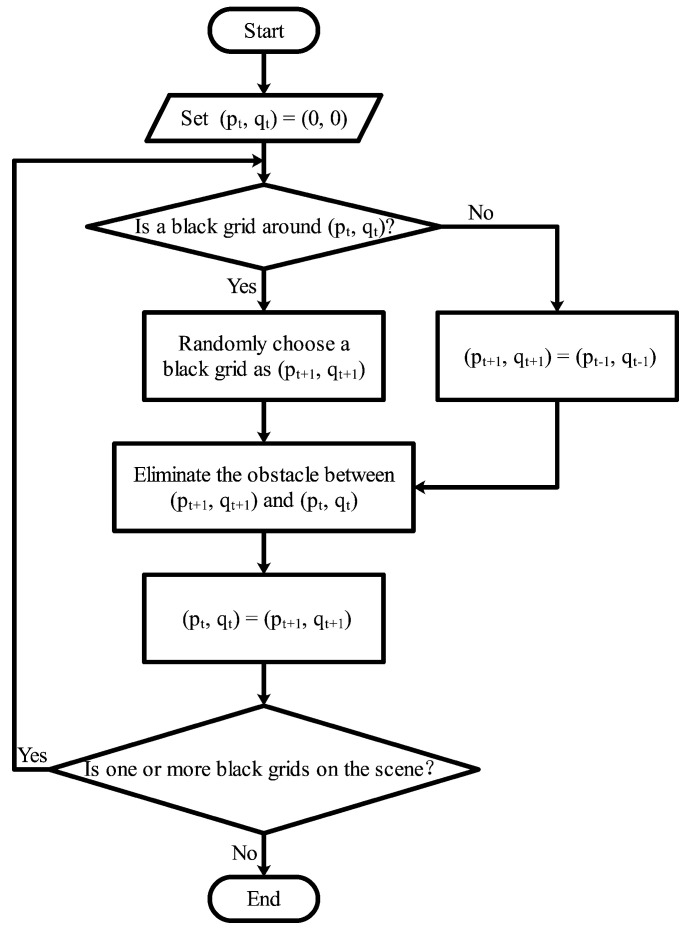
The complete mechanism flow utilized to generate a new maze scene in RoNAP.

**Figure 5:**
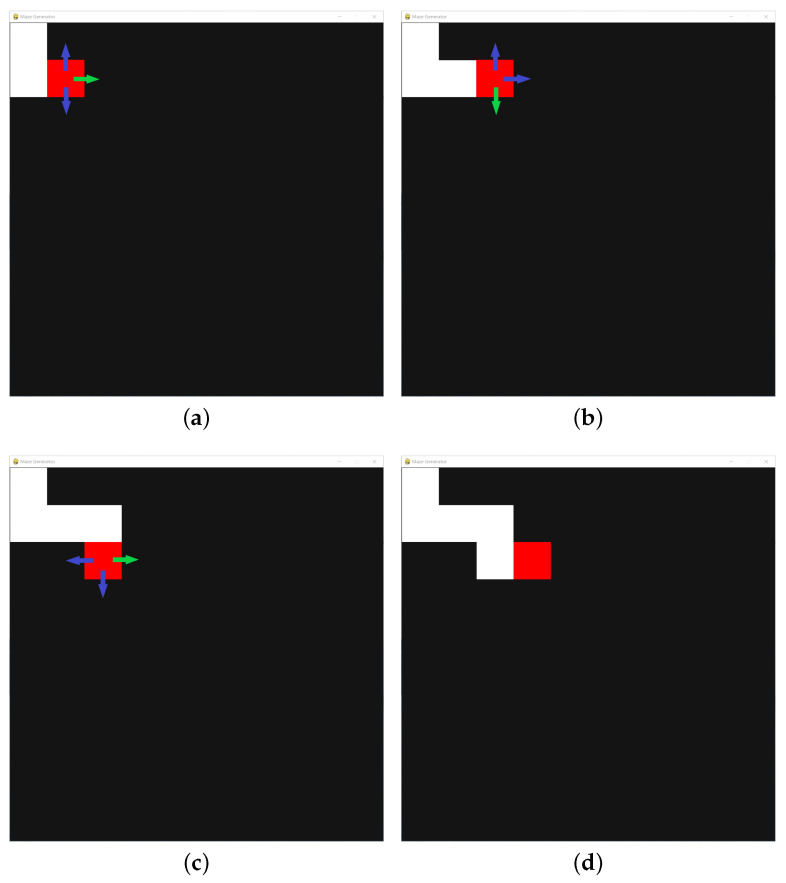
A complete map scene is gradually generated as the red grid moves into the black area: (**a**) step 1, (**b**) step 2, (**c**) step 3, (**d**) step 4. The arrows indicate all possible moving directions, and the green arrow indicates that the direction is randomly selected as the moving direction of the red grid.

The scene of this platform is similar to a maze built by L-shaped obstacles. In order to conveniently change the distribution of obstacles in the maze, a black scene is divided into several square grids. As described in Figure 4, initially, the current position of a red agent $\left(p_{t},q_{t}\right)$ is set on the top first one. A black adjacent grid is randomly selected as the next position $\left(p_{t+1},q_{t+1}\right)$, which turns into a white one after the agent passes by, as shown in Figure 5. Obstacles are placed on four sides of a grid, and the obstacle on the side passed by the red agent is removed, which ensures that there is a channel between any two positions in the scene. If there is no black grid around the agent, it returns to the previous grid and searches for other black grids. The red agent continues to move in the scene until there is no black grid. The grid selected by the red agent is random each time the scene is generated, resulting in different placement of obstacles.

Furthermore, the robot’s movement α is divided into several discrete actions, i.e., turn left, go forward, and turn right. Compared with the continuous action space, the operation of discrete actions is simpler, and their combination allows the robot to move to any possible position in the scene. The robot’s moving speed $\upsilon_{m}$ and rotating speed $\upsilon_{r}$ are set through the **Configurations** button, which allows the user to change the robot’s pose [x,y,z] at each step by

(7) $$\left\{\begin{array}{c} \begin{array}{cc} z=z+\upsilon_{r},\;\;\;\;\;\;\;\;\;\;\;\;\; & \;\;\alpha =turn\;left,\\ z=z-\upsilon_{r},\;\;\;\;\;\;\;\;\;\;\;\;\; & \;\;\alpha =turn\;right,\\ x,\;y=x+\upsilon_{m}\cos z,\;y+\upsilon_{m}\sin z, & \;\;\alpha =go\;ahead. \end{array} \end{array}\right.$$

A user name is set to distinguish different users and facilitate subsequent data search. When this information is set, the initial settings are replaced. A warning alarm “CollisionWarning” appears in case of a collision with obstacles, then the robot returns to the previous position.

The **Collect Data** function is utilized to record human navigation strategies. The robot’s movement is controlled through the keyboard in the scene. The arrow keys on the keyboard correspond to the three discrete actions shown below.
**keyboard**←↑→**action**turn leftgo aheadturn right

To prevent the robot from continuing to move after pressing the key, an anti-mispress function is set such that only after pressing the **down** key can the other arrow keys work. Relevant information is saved in a .csv file in the form shown below.
ρzθDα[498,498…330]135.7367.8410.90AHEAD

Here, ρ is an *m*-dimensional vector representing the distance detected by the laser. As input information, this is sufficient to distinguish the pose of robot in different positions. Moreover, the position of the robot changes randomly after each action through

(8) $$\begin{array}{c} x,\;y,\;z=\lambda_{1}S,\;\;\lambda_{2}S,\;\;\theta +360\lambda_{3}, \end{array}$$

where $\lambda_{1}$, $\lambda_{2}$ are random numbers satisfying a uniform distribution between 0 and 1, $\lambda_{3}$ is a random number satisfying a Gaussian distribution

(9) $$f\left(x\right)=\frac{1}{0.03\sqrt{2\pi }}e^{-\frac{x^{2}}{2\times 0.03^{2}}},$$

and *S* is the size of window. Random change offers the state information of the robot the same probability of being collected, ensuring the integrity and variance of the data. Meanwhile, the random position reduces the continuity of data in both time and space.

RoNAP is user-friendly because it supports a simple form of algorithm access. It is suitable for accessing an input–output model which is established separately on the basis of an algorithm. The established model decides the robot’s action according to the observed scene information. This platform changes the position of the robot in the scene based on the executed action, and the new state information is saved and passed to the model as the next input. Looping continues until the end of the task. In addition, there is no need for complicated operations in the process of algorithm testing; the only step is to simply click the corresponding button according to the prompts.

The above functions are only the initial settings of RoNAP; the outstanding feature of the platform is that its parameters can be changed according to the user’s needs. The functions of the platform are written in different modules using Python, meaning that users who know programming can easily find the code corresponding to each function. Taking the action of robots as an example, it can be set to a combination of angular velocity and linear velocity. The only change to the code that is needed is the way in which the robot’s coordinates are calculated after performing an action. Other parameters can be modified by modifying the code as well, such as the number of lasers, the maximum detection distance, the size of scene, etc.

## 3. Instructions for Using RoNAP

RoNAP is a lightweight navigation analysis platform based on Python packages, and can be stably run on personal computers without high-performance hardware support. In this section, detailed instructions about the use of RoNAP are introduced.

### 3.1. Running Environment of RoNAP

The analysis platform is completely built using Python code, which is an object-oriented high-level programming language. All files are placed in a folder, where users directly run the file named “main.py” to start RoNAP with any editor. To successfully start the platform, the user’s computer needs to be configured with a Python environment, and the version must be above 3.6. Due to the light weight of RoNAP, it only needs a CPU to implement the simulation process, with GPU accelerating rendering being unnecessary.

The file named “main.py” is the main program of the whole project, in which several parameters are defined, such as the size of the maze and obstacles, background, color, etc. Each parameter definition is attached with comments to help users modify it according to their own requirement. Customized functions are included in this file as well to make the program clearer. In the initial setup, a neural network is employed as an input–output model to guide the robot’s movement. It can be replaced with other models in the file named “Network_train.py”, although note that the input and output interfaces are designed to match the original model, which avoids the need for large program modifications. The visual interface design of the whole platform is integrated in the file named “ScreenFunction.py”. Each visual interface is encapsulated as an application programming interface (API), which is directly called to present the corresponding interface. The file named “MazeClass.py” defines a class responsible for maze generation. A random parameter is set in this file to generate different scenes without modifying the code. These individual files form a project to support the stable operation of the platform.

### 3.2. Operation Process of RoNAP

Before performing any analysis on RoNAP, the movement parameters of the robot need to be set through the function button **Configurations**. In this function, the movement speed, rotation speed, and user name are set according to the text written in the boxes. Note that the movement speed must be less than 5 to avoid the mobile robot traversing obstacles in one step. It is best not to set the rotation speed too small in order to avoid the mobile robot rotating in place for a long time. A user name is set to differentiate different users in order to ensure that personal data is stored separately with respect to its name. Clearly, as an open source platform, basic settings can be modified in the program, such as color, robot style, various sizes, etc.

A qualified navigation algorithm has the ability to cope with changes in the scene. Therefore, a variety of map scenes must be included in an analysis platform. In RoNAP, the distribution of obstacles is changed through the function button **Maze Generator** before the analysis. The newly generated scene is saved as a picture named “maze”, which is used in the subsequent navigation analysis. The program sets a start position and a destination point randomly by default, and they can be set to fixed values to keep each analysis process invariant.

Loading an input–output model into the mobile robot, the change in its movement is observed through the function button **Start** in the platform. At each time step, the color of each laser and obstacles within the detection range can be observed clearly. Pressing the *M* key while the program is running shows the path that the robot traverses, indicated by a blue line in the whole scene.

The information of the robot is recorded at each time step and stored in a .csv file through the function **Collect Data**, which can be found in the same folder. The .csv file is easy to read and process with a Python compiler. Additionally, the developer’s information is described by clicking on the **Developer** button.

## 4. Mobile Robot Navigation Approaches Analysis Based on RoNAP

Current robot navigation approaches are mainly divided into the following two types: one is local path planning, in which environmental information is partially or completely unknown, and the other is global path planning, in which all environmental information is known. In this section, several different types of mobile robot navigation approaches, such as Reinforcement Learning [24], Deep Learning [25], and the Artificial Potential Field Method, are tested in RoNAP, proving that this platform has the ability to support simulation and analysis of various input–output model-based navigation algorithms.

### 4.1. Problem Formulation

**Figure 6:**
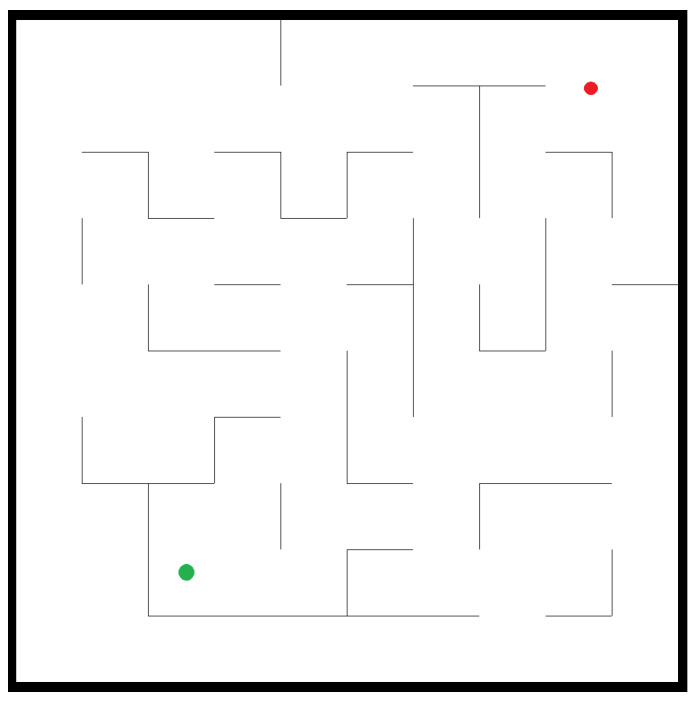
The 2D simulation environment in RoNAP, with the start position, destination position, and obstacles all set randomly. The red dot is the start position and the green dot is the destination position.

In RoNAP, the 2D working scene of a mobile robot is created in the form of a maze in which L-shaped obstacles are generated randomly, as shown in Figure 6. In this figure, the red dot is denoted as the start position $\left(x_{0},\;y_{0}\right)$, while the green dot is denoted as the destination position $\left(x^{d},\;y^{d}\right)$. In this platform, the navigation task is defined as below.

**Definition** **1.** *The mobile robot autonomously moves from $\left(x_{0},\;y_{0}\right)$ to $\left(x^{d},\;y^{d}\right)$ and no collisions occur during the whole process.*

This platform is suitable for solving the navigation task using an input–output model which perceives the surrounding environment information and outputs a correct action. Several pieces of information mentioned in Section 2.2 are extracted as input information, which is necessary in order to distinguish the pose of the robot in different states. The change in the robot’s position is observed through the running interface. The connection of all positions forms a complete path, which is used to determine whether to complete the task defined in Definition 1.

### 4.2. Deep Q-Learning Network Algorithm

The Deep Q-learning network (DQN) is a reinforcement learning algorithm which combines the decision-making capacity of a Q-learning algorithm and the high-dimensional representation capability of a neural network. As a local navigation approach, DQN employs a neural network to fit complex value functions, which outputs the value of each action in real-time according to the observed environmental state. The action with the highest value is selected to be executed at each time point.

As described in Algorithm 1, the mobile robot perceives the surrounding environment through sensors, the action with highest value is executed, and the reward function provides a value judgment [26]. The current state $s_{t}$, action $a_{t}$, reward $r_{t}$, and next state $s_{t+1}$ are recorded into an experience pool, from which a batch of data is randomly selected to train the neural network. The parameters of the neural network are constantly adjusted through the gradient descent algorithm, resulting in changes in the value of each action. Through a procedure of continuous iterative learning, the neural network finally converges to the optimal result in a certain episode when it is capable of making a correct value evaluation for each action under any condition.

Inadequate or sparse rewards frequently lead to the poor efficiency of learning strategies. Therefore, choosing a suitable reward function is crucial to the success of the algorithm. This paper sets the reward function as follows:

(10) $$r=r_{a}+r_{b}+r_{c}+r_{d},$$

where the notations used above are listed below:

$r_{a}$ is a collision penalty function set to reduce collisions with obstacles
$r_{b}$ is a distance reward function set to prompt the robot to move toward the destination position
$r_{c}$ is a turning penalty function set to avoid the robot spinning around
$r_{d}$ is a destination reward function set to issue a reward for completing the task

**Algorithm 1** DQN training procedure.**Input:**  Total training episodes $\Gamma_{max}$, experience pool capacity *D*, target network update frequency *F*, training batch size *B*, attenuation coefficient γ, greedy value ε and maximum total penalty $R_{min}$.**Output:**  Target neural network $\hat{Q}$ with $\hat{\omega }$.1: **Initialization:** set initial episode Γ=0, initial state $s_{t}$, total reward of each episode R=0, value neural network *Q* with random weight ω, target network $\hat{Q}$ with $\hat{\omega }=\omega$.2: **while**
$\Gamma <\Gamma_{max}$
**do**3: **while**
$∥\left(x,\;y\right)-\left(x_{d},\;y_{d}\right)∥⩾\epsilon$

   
$R⩾R_{min}$

**do**
4:  Select an action through $a_{t}=argmaxQ\left(s_{t},a_{t};\omega \right)$.5:  Execute $a_{t}$ and obtain $r_{t}$ and $s_{t+1}$.6:  Store $\left(s_{t},\;a_{t},\;r_{t},\;s_{t+1}\right)$ into *D*.7:  Randomly select *B*-size data from *D*, and perform
(11)$$y=\left\{\begin{array}{cc} r_{t}, & \Gamma =\Gamma_{max},\\ r_{t}+\gamma max\hat{Q}\left(s_{t+1},a_{t+1};\hat{\omega }\right), & \mathrm{else}. \end{array}\right.$$8:  Compute mean square error loss through (12)$$loss={\left(y-Q\left(s_{t},\;a_{t};\;\omega \right)\right)}^{2}.$$9:  Utilize gradient descent algorithm to update ω.10:  After *F* steps, perform $Q=\hat{Q}$.11:  Perform $R=R+r_{t}$.12: **end while**13: Perform Γ=Γ+1 and R=0.14: **end while**15: **return** target neural network $\hat{Q}$ with $\hat{\omega }$.

Analyzing the DQN algorithm on this platform successfully benefits from its abundant sensors. In this paper, 17 lasers are installed to obtain the distance $d_{i}\;\left(i=1,2\ldots 17\right)$ between the robot and the surrounding obstacles to achieve obstacle avoidance. The symbol ϕ is relevant forward angle, and is computed by

(13) ϕ=|z−θ|,

which is utilized to guide the robot towards the destination. The state of the mobile robot treated as the input of the model is described by an 18-dimensional vector

(14) $$z_{0}^{in}={\left[d_{1}\;d_{2}\ldots \;d_{17}\;\phi \right]}^{⊤}.$$

This allows the neural network to accurately distinguish the state of the robot.

**Figure 7:**
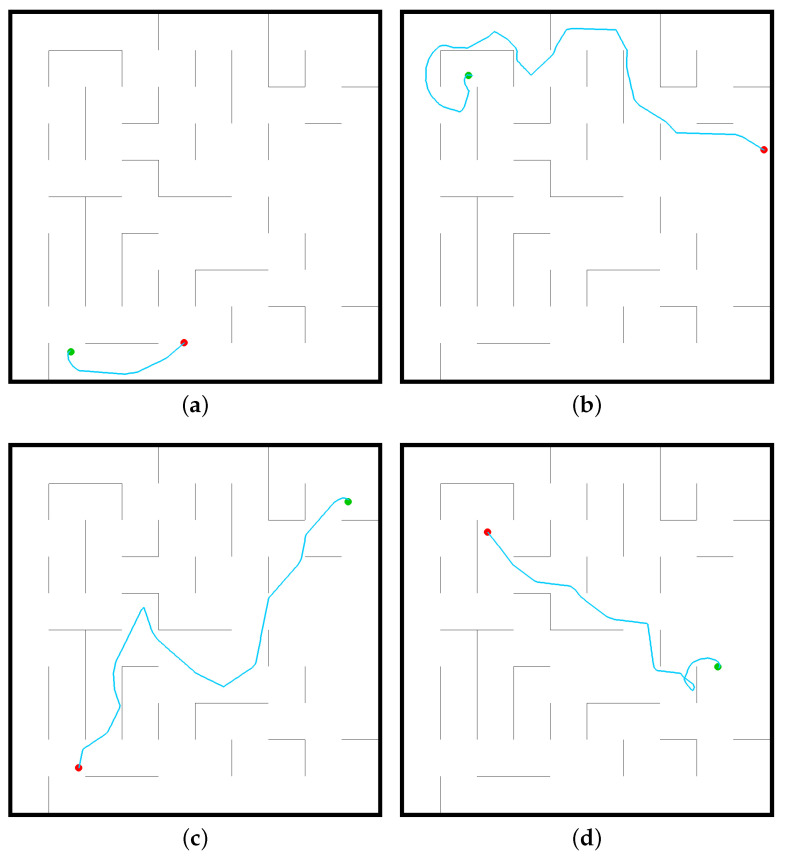
Successful cases using the DQN algorithm to solve the mobile robot navigation task by analysis in RoNAP: (**a**) DQN_case1, (**b**) DQN_case2, (**c**) DQN_case3, (**d**) DQN_case4.

The DQN algorithm is a process of constantly optimizing the neural network. RoNAP provides an interface in which the movement tendency of robot is observed during training, which is convenient for making modifications targeted to specific issues. Through this platform, it is obviously observed that the robot moves illogically at first, then it consciously moves towards the destination point as the amount of training increases. When the mobile robot is able to move continuously towards the destination without collisions, as shown in Figure 7, this shows that the neural network is capable of making correct decision autonomously based on the observed information.

### 4.3. Deep Learning Algorithm

Deep learning (DL) is an important field in machine learning, and has significant applications in natural language processing [27], image segmentation [28,29], computer vision [30], fault diagnosis [31], and performance optimization [32]. Similar to the DQN algorithm, DL algorithms [33] train a neural network to output correct actions according to the surrounding environment information. The difference is that deep learning mainly relies on human data collected in advance to train a neural network that is able to replicate human behaviors and does not require the agent to explore the scene. DL approaches are mainly divided into a training phase and a prediction phase. The training phase extracts part of the data for use as training data to train a neural network through gradient descent, in which the parameters are adjusted to minimize the loss function. The prediction phase predicts the outputs of the remaining input data and observes whether it conforms to the labels. When the accuracy of replicating human behavior reaches the desired value, a robot loaded with the trained neural network is theoretically capable of performing autonomous navigation. The specific algorithm flow is shown in Algorithm 2.

**Algorithm 2** DL training procedure.**Input:** Data set Λ, training data number $n_{train}$, training batch size *B*, total training epoch number $\Gamma_{max}$ and learning rate lr.**Output:** A neural network $\hat{Q}$ with $\hat{\omega }$.1: **initialization:** Set the first training epoch number Γ=1 and a neural network *Q* with random weight ω.2: Randomly select $n_{train}$ data from Λ as $\Lambda_{train}$ and the rest to testing data set $\Lambda_{test}$.3: **while**
$\Gamma <\Gamma_{max}$
**do**4:  Randomly select *B* data from $\Lambda_{train}$, and compute the sum of the cross entropy loss.5:  Compute the gradients of ω.6:  Perform Adaptive Moment Estimation (Adam) gradient descent training to update ω using the obtained gradients.7:  Calculate the accuracy of neural networks replicating human behavior in training data set $\Lambda_{train}$.8:  Perform Γ=Γ+19: **end while**10: Calculate the accuracy of neural networks replicating human behavior in training data set $\Lambda_{test}$.11: **return** the trained neural network $\hat{Q}$ with $\hat{\omega }$.

**Figure 8:**
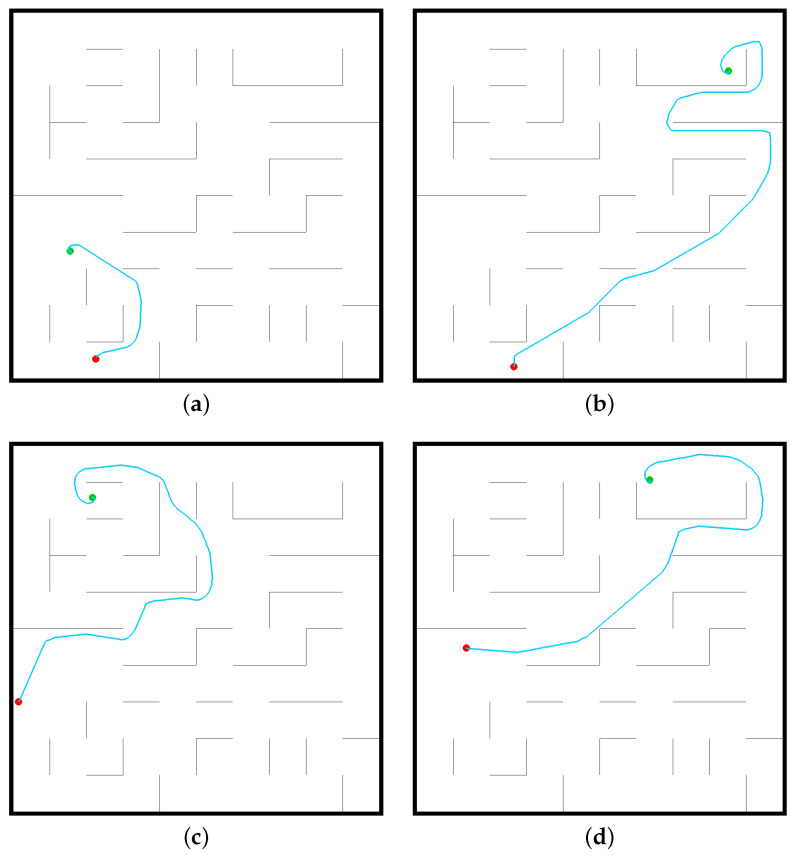
Successful cases using a DL algorithm to solve the mobile robot navigation task through analysis in RoNAP: (**a**) DL_case1, (**b**) DL_case2, (**c**) DL_case3, (**d**) DL_case4.

In deep learning, the reliability of the data is the key to success; however, there is a lack of authoritative public datasets in common applications. Therefore, researchers have to record relevant data by themselves for different cases. The RoNAP approach proposed in this paper supports a data collection function, **Collect Data**. The input and label information are recorded in a file that is easy to read and use to train a neural network. The trained network then makes correct decisions based on the input information in order to lead the robot to the destination, as shown in Figure 8.

### 4.4. Artificial Potential Field Method

The artificial potential field (APF) method is a global path planning algorithm. It aims to abstract the scene into an artificial force potential field, in which the destination position generates gravitational force and the obstacle generates repulsive force on the robot. The repulsive force is inversely proportional to the distance to the obstacle, while the gravitational force is proportional to the distance to the destination. The result of both forces controls the movemnt direction of the robot.

In this paper, the force potential energy at each position is set as follows:

Gravitation potential energy is calculated by

(15) $$U_{grav}=\frac{1}{2}\xi d^{2}\left(q,q_{goal}\right),$$

where ξ is the gravitation gain and $d(q_{1},q_{2})$ is the distance between two positions;

Repulsion potential energy is calculated by

(16) $$U_{repu}=\left\{\begin{array}{c} \frac{1}{2}\eta \frac{1}{d^{2}\left(q,q_{obs}\right)},\;\;d\left(q,q_{obs}\right)⩽d_{max},\\ 0,\;\;d\left(q,q_{obs}\right)>d_{max}, \end{array}\right.$$

where η is the repulsion gain;

Resultant force is calculated by

(17) $$U=U_{grav}+U_{repu}.$$

As shown in Algorithm 3, when *U* is used to represent the total potential energy of each position, the gradient ∇U represents the force vector at this position. The opposite direction of the gradient at a certain position is the direction in which the potential function drops the fastest. The artificial potential field method is started as the robot starts from the initial position and walks along the opposite direction of the gradient until arriving at the destination position, where the gradient is 0.

**Algorithm 3** APF method implementation procedure.**Input:** Scene map maze, random start position $\left(x_{0},y_{0}\right)$, random destination position $\left(x^{d},y^{d}\right)$, positions set $\Lambda_{positions}$.**Output:** A path between $\left(x_{0},y_{0}\right)$ and $\left(x^{d},y^{d}\right)$.1: **initialization:** Set current position $\left(x,y\right)=\left(x_{0},y_{0}\right)$, gray processing and binarization for the map maze.2: Confirm the location of obstacles and calculate potential energy of each position.3: **while**
$|\left(x^{d},y^{d}\right)-\left(x,y\right)|>\epsilon$
**do**4:  Record current position into $\Lambda_{positions}$.5:  Find the position (x,y) with the smallest potential energy among the eight positions around (x,y).6:  Perform $\left(x,y\right)=\left(x_{t+1},y_{t+1}\right)$.7: **end while**8: **return** A path with all positions in $\Lambda_{positions}$.

The artificial potential field method is able to be simulated on this platform mainly due to the **Maze Generator** function. The scene map is loaded as a picture, allowing the position of each scene element to be obtained accurately, which contributes to subsequent potential energy calculations. In order to facilitate the judgment of obstacles, the picture is first processed in grayscale, with each pixel is represented by a number between 0 and 255; then, the map is binarized as

(18) $$\tau_{\left(x,y\right)}=\left\{\begin{array}{c} \begin{array}{cc} 0, & \;\tau_{\left(x,y\right)}⩽\tau_{thresh},\\ 255, & \;\tau_{\left(x,y\right)}>\tau_{thresh}, \end{array} \end{array}\right.$$

where $\tau_{\left(x,y\right)}$ is the gray value of each position and $\tau_{thresh}$ is the threshold. In this way, the entire map is composed of black obstacles and white movable space.

**Figure 9:**
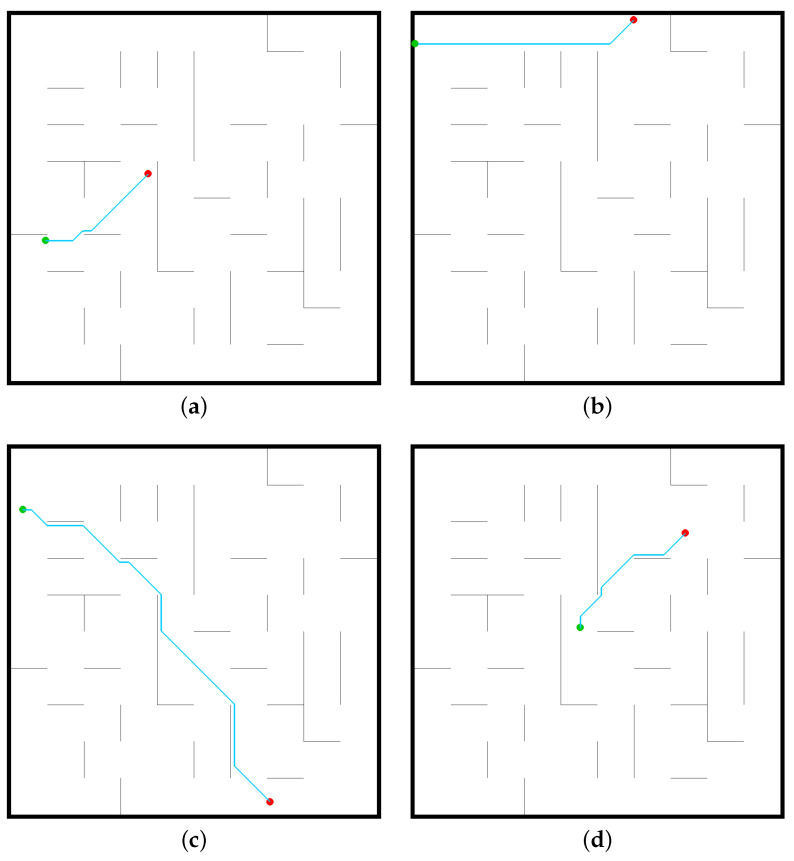
Successful cases using the APF algorithm to solve the mobile robot navigation task through analysis in RoNAP: (**a**) APF_case1, (**b**) APF_case2, (**c**) APF_case3, (**d**) APF_case4.

In the artificial potential field method, the potential energy of each position is calculated according to different formulas. The input–output model is a matrix containing the potential energy information of all positions. The current coordinate of the robot (x,y) in the scene is regarded as the input information of the model, and the position of the 8 neighbors with the smallest potential energy is chosen as the next moving position until the destination position is reached. The robot moves to the next position through a combination of discrete actions. Successful analysis results using APF method are shown in Figure 9.

## 5. Conclusions and Future Work

This paper introduces a self-developed mobile robot navigation analysis platform named RoNAP based on Python. It has the following outstanding features. First, RoNAP constructs a scene similar to a maze, which is very similar to a modern logistics warehouse. In this scene, the L-obstacles, start position, and destination position are all set randomly. The robot is required to move from the start position to destination position without colliding with any obstacles. This requirement simulates the task of transporting goods to a designated location safely. Second, various devices are provided in RoNAP which are sufficient to perceive the surrounding environment, including laser sensors, gyroscopes, and radar locators. In addition, RoNAP provides a clear interface in which the pose of the robot and the path it moves along can be observed in real time. Third, in addition to being an algorithm analysis platform, RoNAP can be used to complete data collection tasks. It provides accurate data for researchers who want to use neural networks to solve navigation problems. Fourth, compared with existing analysis platforms, its advantage lies in its simple operation. The only step required during the whole analysis process is to click the corresponding buttons. Moreover, this platform does not require powerful computer hardware to support, and the analysis speed is fast. Fifth, the functions of RoNAP are written in blocks using Python language, which allow users to modify relevant parameters according to the needs of different algorithms. Three types of algorithms have been tested on this platform, and all achieved the desired results. The whole test was performed for more than 10,000 simulation operations, proving the stability and reliability of RoNAP. Lastly, all the code used in the platform is open-source and has detailed comments attached. In the developer mode, it is convenient for users to modify the underlying code to complete the analysis and testing of personal algorithms.

This platform is mainly used to simulate a real-world storage environment. In the future, more navigation algorithms can be tested. Multi-robot collaboration is an effective means of improving storage efficiency; thus, a function that allows for communication between multiple robots could be considered for application on this platform. In addition, the application scenarios of robots are becoming more and more complex, and it is crucial to make real-time decisions based on the specific scenario. Thus, more scene elements should be added, for example, obstacles appearing randomly during robot movement and multi-destination optimal path planning, which is more in line with the actual tasks encountered in the logistics industry. Additionally, more mode options should be developed so that users do not have to modify the code themselves, which would make the platform more friendly to programming novices. Accounting for these potential improvements, the current version of RoNAP is nonetheless sufficient to support the simulation analysis of multiple navigation methods with high processing speed and low cost, and the underlying algorithm can be further optimized to improve the platform’s suitability and sustainability.

## Data Availability

Not applicable.

## Abbreviations

The following abbreviations are used in this manuscript:

RoNAP	Robot navigation analysis platform
GPU	Graphics processing unit
CPU	Central processing unit
DQN	Deep Q-learning network
DL	Deep learning
APF	Artificial potential field
